# Cerebellar Neurostimulation for Boosting Social and Affective Functions: Implications for the Rehabilitation of Hereditary Ataxia Patients

**DOI:** 10.1007/s12311-023-01652-z

**Published:** 2024-01-25

**Authors:** Andrea Ciricugno, Viola Oldrati, Zaira Cattaneo, Maria Leggio, Cosimo Urgesi, Giusy Olivito

**Affiliations:** 1 IRCCS Mondino Foundation, 27100 Pavia, Italy; 2 Department of Brain and Behavioral Science, University of Pavia, 27100 Pavia, Italy; 3 Scientific Institute, IRCCS Eugenio Medea, 23842 Bosisio Parini, Italy; 4 Department of Human and Social Sciences, University of Bergamo, 24129 Bergamo, Italy; 5 Department of Psychology, Sapienza University of Rome, 00185 Rome, Italy; 6 Ataxia Laboratory, Fondazione Santa Lucia IRCCS, 00179 Rome, Italy; 7 Laboratory of Cognitive Neuroscience, Department of Languages and Literatures, Communication, Education and Society, University of Udine, 33100 Udine, Italy

**Keywords:** Cerebellum, Social cognition, Emotion, Brain stimulation, Hereditary ataxia

## Abstract

Beyond motor deficits, spinocerebellar ataxia (SCA) patients also suffer cognitive decline and show socio-affective difficulties, negatively impacting on their social functioning. The possibility to modulate cerebello-cerebral networks involved in social cognition through cerebellar neurostimulation has opened up potential therapeutic applications for ameliorating social and affective difficulties. The present review offers an overview of the research on cerebellar neurostimulation for the modulation of socio-affective functions in both healthy individuals and different clinical populations, published in the time period 2000–2022. A total of 25 records reporting either transcranial magnetic stimulation (TMS) or transcranial direct current stimulation (tDCS) studies were found. The investigated clinical populations comprised different pathological conditions, including but not limited to SCA syndromes. The reviewed evidence supports that cerebellar neurostimulation is effective in improving social abilities in healthy individuals and reducing social and affective symptoms in different neurological and psychiatric populations associated with cerebellar damage or with impairments in functions that involve the cerebellum. These findings encourage to further explore the rehabilitative effects of cerebellar neurostimulation on socio-affective deficits experienced by patients with cerebellar abnormalities, as SCA patients. Nevertheless, conclusions remain tentative at this stage due to the heterogeneity characterizing stimulation protocols, study methodologies and patients’ samples.

## Introduction

### The Cerebellar Role in Socio-Affective Functions

Over the last decades, a consensus has been reached about the role of the cerebellum in affective and social functions [1, 2] and increasing evidence has emerged about its inclusion in the cortico-limbic networks subserving emotion processing [1, 3, 4]. Indeed, emotional processing is considered one of the main components of social cognition [5], defined as a set of mental processes engaged by humans to comprehend, produce, and regulate social behavior to interact with others in a social environment [6, 7]. A fundamental aspect of social cognition is Theory of Mind (ToM), or the “mentalizing” process, i.e. the ability to attribute mental states (such as emotions, intentions, and beliefs) to others to explain and predict their behavior [8, 9].

The cerebellar connectional and functional topography provides the critical anatomical substrate [10] to understand the functions of the cerebellum, including its role in social cognition. It is commonly assumed that the cerebellum operates as a co-processor of a wide range of functions, by modulating the activity of key cerebral regions to which different cerebellar modules are connected [11]. This cerebro-cerebellar connectivity thus affects sensory-motor processing as well as cognitive and affective functions [2, 10].

More in general, the sequence detection theory [12] suggests that the cerebellar operational model is the same regardless of whether the information to be processed is sensory-motor, cognitive, or behavioral. According to this model, the cerebellum detects and memorizes patterns by constructing internal models of the experienced sequence of events. This predictive and sequential coding can be also extended to social behavior [2, 13, 14], and emotion regulation [15]. The idea is that the cerebellum may modulate cerebral activity to promote the correct implementation of social action sequences, and to adjust unexpected events when violations from predicted scenarios are met [12].

According to the “dysmetria of thought” hypothesis by Schmahmann and colleagues [16], cerebellum-related affective and cognitive deficits would mirror diminished (hypometric) or exaggerated (hypermetric) responses to the internal and/or external environment [17]. In this view, cerebellar structural alterations may affect the modulatory function of the cerebellum on the cortical projection areas involved in emotional and social processing, so that behavior is not always appropriately adjusted to specific social environmental requirements [2, 14]. This interference may lead to specific impaired social outcomes, particularly when the specific situation/interaction requires advanced ToM abilities and a high level of prediction.

Social cognition and high-level ToM functions require complex interactions between limbic, associative, and subcortical areas [18–21]. ToM abilities seem to mainly depend on a group of brain regions, called the “mentalizing network,” which includes regions in the superior temporal sulcus (STS), temporoparietal junction (TPJ), medial precuneus, and dorso medial prefrontal cortex (dmPFC, [22, 23]).

It is widely acknowledged that the cerebellum is incorporated into associative and paralimbic circuits involved in affective and social processes [24, 25]. The cerebellar involvement in the social brain network has been widely supported by resting-state fMRI studies. Indeed, the investigation of functional connectivity has identified a cerebellar topography for social functions showing neural synchronization between distinct cerebellar and cerebral zones, known to be strictly related to affective functions and social mentalizing [26, 27]. Functional coherence has been found between the cerebellar vermis and brain limbic structures typically implicated in emotional regulation [28], such as the hippocampus, involved in memory and learning processes [29], and the amygdala, known to modulate distinct aspects of emotional processes [30]. The cerebello-cerebral network related to the most abstract and complex forms of mentalizing has been specifically characterized by a multi-study analysis of Van Overwalle and Mariën [21] and included the dmPFC, precuneus/ posterior cingulate cortex, bilateral TPJ, and a region in the posterior cerebellum corresponding to the right Crus II. Overall, these observations suggest that some areas of the cerebellum may be preferentially recruited for specific components of social mentalizing.

While many functional studies showed cerebellar activations during emotional processing and mental state inference tasks [31, 32], clinical studies provided further support to the view of a “social cerebellum” reporting an impaired performance of patients with cerebellar damage in a range of perceptual [33], affective, cognitive [34, 35], and ToM tasks [36] that are essential in social interactions. The prevalent idea is that these impairments may be caused by abnormal cerebellar modulation of cerebral areas involved in emotional and mentalizing processing, such as limbic, frontal, and temporo-parietal areas [21, 37, 38].

### Social and Affective Disturbances in Hereditary Ataxia

Increasing evidence suggests the occurrence of emotional and social disturbances in patients with various types of cerebellar diseases, affecting the quality of their social life [14, 39, 40]. In the context of cerebellar disease, the spinocerebellar ataxias (SCAs) are a group of rare (prevalence rate of 1–4 in 100,000) [41] neurodegenerative disorders of autosomal dominant inheritance resulting from degeneration of the cerebellum and its connections. Many studies demonstrated the presence of cognitive [42, 43] emotional and neuropsychiatric disorders in these pathologies [35, 44] grouped in the so-called “cerebellar cognitive affective syndrome” (CCAS) [34].

Starting from these observations, an increasing body of studies has reported that SCA patients also present alterations in various aspects of social cognition, from the perception of emotions to ToM [45–47]. The results about the pattern of observed impairments, however, have been conflicting [48]. Some authors suggest that the cerebellum may be exclusively involved in more complex aspects of social cognition, showing an impairment of ToM abilities in patients with SCA3 and SCA6 [49], whereas the attribution of basic emotions evaluated by verbal tasks would be spared. However, other studies reported that SCA2 and SCA7 patients are impaired also in verbal emotion attribution tasks, thus suggesting that social cognition impairment in SCA patients is not homogeneous among the various genotypes [47]. Authors showed that patients with SCA2 and SCA7 present difficulties in attributing emotions, such as happiness, sadness, fear, anger, and embarrassment, corroborating previous findings of cerebellar involvement in emotional processing [47], (for a review see also [1]). In the study of Sokolovsky and colleagues [47], no emotional impairment was reported in SCA1 patients, while a ToM impairment was described in only one SCA1 patient, with no evidence of this deficit in any of the SCA2 or SCA7 patients. According to the described findings [47, 49], the distinct impairment profiles of the five patient groups (SCA1, SCA2, SCA3, SCA6, and SCA7) can be explained by the segregation of functions within the cerebellum.

A later study by D’Agata and colleagues (2011) showed that emotion recognition, as assessed by the Ekman 60 Faces battery and the Tamietto 50 Faces test [50, 51], is impaired in patients with hereditary ataxia of different genotypes, supporting the evidence previously reported in SCA2 and SCA7 and adding evidence of emotion recognition impairment in SCA6 and SCA8 patients. In particular, this study indicated that SCA patients have a prominent deficit in the identification of more complex social emotions, both positive and negative, with respect to basic emotions [45]. These findings are supported by another study using the revised Reading the Mind in the Eyes (RMET) [52] showing that both patients with complex cerebrocerebellar degeneration (i.e. SCA1, SCA2, SCA7, SCA17) and those with an isolated cerebellar disease (i.e. SCA3, SCA6, episodic ataxia type2) were impaired in emotion attribution and in processing negative and positive emotions compared to emotionally neutral stimuli [46]. These findings are in line with the evidence that the cerebellum participates in the complex network that processes emotional stimuli, especially those having an emotionally negative valence [53, 54].

In terms of underlying neural substrate, neuroimaging studies have supported the relationship between structural and functional cerebellar alterations and the impairment of different social cognition abilities in different cerebellar patients [13, 39, 40]. The gray matter (GM) reduction in specific portions of the cerebellum (vermis and bilateral Crus I/II) has been linked to social impairment in patients affected by cerebellar neurodegenerative pathologies [13]. Intriguingly, these areas showed decreased functional connectivity with cerebral areas involved in mirroring and mentalizing processing [20, 21].

Altered cerebello-cerebral functional coupling has been also related to social impairment in patients with hereditary ataxia. Aberrant inter-nodal functional connectivity between the posterior cerebellum and cerebral regions related to social cognition processing was recently found in a homogenous population of patients affected by SCA2 [40]. These results suggest that the atrophy of specific cerebellar portions and disruption of the cerebello-cortical pathway may subtend the social cognition deficits in SCA2. Consistently, a more recent study provided an extensive characterization of the social cognition profile of SCA2 patients, who showed impairment in the immediate perceptual component of the mental state recognition (i.e., recognizing feelings and thoughts of other people from eye expressions) and difficulties in understanding false or mistaken beliefs as assessed by the RMET [52] and Faux Pas [55] tests, respectively. Interestingly, the authors found that patients’ performance on each impaired task correlated with specific MRI changes. A direct correlation was found between alterations in more complex components of social mentalizing, as assessed by the Faux Pas, and GM volumes in the right Crus II [39].

Further evidence comes from another recent study showing that patients with hereditary (SCA1, SCA2) and idiopathic ataxia exhibit impaired performance on both the Faux Pas Recognition Test and the RMET [56]. Patients have difficulty in understanding the mental states of others in everyday interactions and from their facial expressions.

Overall, the present results suggest that social cognition presents both typical and specific alterations according to the SCA variant [57]. The characterization of social and emotional features in different SCA subtypes may help the management of patients’ quality of life and could serve as a possible preclinical marker of the disease. Most importantly, the present findings have opened a substantial body of studies investigating the modulating effects of cerebellar stimulation on social skills. This may have important implications in the clinical and translational field to consider the cerebellum as a potential neurostimulation target across multiple pathological conditions.

### Non-Invasive Brain Stimulation

Non-Invasive Brain Stimulation (NIBS) techniques are widely used in healthy adults to investigate brain mechanisms or to modulate and enhance cognitive and socio-affective processes [58]. NIBS techniques, both transcranial magnetic stimulation (TMS) and different forms of transcranial electrical stimulation, including transcranial direct current stimulation (tDCS), are used to boost neuropsychological or psychiatric rehabilitation, through modulation of neuroplasticity. In TMS protocols, a coil placed above the scalp delivers a brief and high-amplitude current and generates a magnetic pulse that induces a transitory electric current in the cerebral surface under the coil. With sufficient intensity, a single pulse of TMS causes highly synchronized action potentials in the targeted area. TMS can be delivered as a single-pulse, repetitive (i.e., rTMS, series of pulse trains) or in a patterned fashion, such as theta-burst stimulation (TBS), in which a series of pulses are delivered in bursts of high frequency (i.e., 50 Hz) with an interburst interval of 200 ms (i.e., 5 Hz). TMS represents a powerful tool for investigating causal brain-behavior relations complementing correlative techniques such as functional neuroimaging. Indeed, if stimulating a cortical region significantly affects task performance, this indicates that the targeted area is necessary to perform the task normally. Therefore, TMS effects have traditionally been interpreted to interfere with brain function, by inducing a transient, reversible “virtual lesion” in the targeted region, with impairment as its default outcome [59, 60]. TMS could also enhance brain activity and behavioral performance (for a review, [61]). Indeed, TMS behavioral effects are state-dependent, and factors such as stimulation parameters, task difficulty, and cognitive state can fundamentally change the stimulation outcome [62–64]. For instance, rTMS-induced effects are frequency-dependent, with low (≤ 1 Hz) and high frequencies (≥ 5 Hz) decreasing and increasing cortical excitability, respectively (for a review, [65]. As for TBS, continuous TBS (i.e., cTBS), in which bursts of pulses are delivered without interruption, reduces cortical excitability, while intermittent TBS (i.e., iTBS), in which short intervals separate bursts of pulses, enhances it. TMS has a relatively good spatial resolution, (see [66] for more details) and a high temporal resolution (see [67, 68] for examples). TMS can be applied using either an online or an offline protocol. In online protocols, either single pulses or short trains of pulses (typically delivered at 10 or 20 Hz) are delivered while individuals are engaged in a task (for review, [69]). In offline paradigms, task performance is assessed before and after the stimulation, during which a series of pulse trains are applied over a period typically lasting 10 to 20 min, with stimulation aftereffects on behavioral performance outlasting the period of stimulation by many minutes or hours (depending on the stimulation protocol and its parameters).

In tDCS protocols, the current is typically delivered using a bipolar montage consisting of the active electrode (anode or cathode, depending on the experimental design) located directly over the targeted region and the reference electrode located over either a cephalic site (commonly, the contralateral supraorbital region) or an extracephalic site (e.g., the deltoid or the buccinators muscles). tDCS does not directly induce cerebral activity, but it rather alters spontaneous brain excitability by subthreshold modulation of the neural resting state potential [70]. Currently, tDCS devices apply a weak direct electrical current (0.5–2 mA), typically for a relatively long period of time (e.g., 20 min). Depending on the electrode polarity, the stimulation facilitates (anodal) or inhibits (cathodal) spontaneous neuronal activity resulting in modulation of neuronal excitability and neuroplastic reorganization [70]. However, as for TMS, physiological and behavioral tDCS-induced effects depend on a complex interaction between stimulation parameters and endogenous neural activity (e.g., [71, 72]). This aspect is particularly relevant when stimulating the cerebellum. Indeed, given its entirely different cytoarchitecture compared to the neocortex, the cerebellar cell morphology, and the complex cerebellar folding, when applied to the cerebellum, tDCS polarity is not predictive of the direction of the behavioral changes (for a meta-analysis see [73]). Similarly to TMS, tDCS can be administered either online or offline (see [74]). Ten minutes or more of tDCS can lead to modulatory effects that outlast the period of stimulation by many minutes or hours, with more robust behavioral effects being detectable immediately after the end of the stimulation (for reviews, [75]).

### Aim of the Present Scoping Review

This scoping review aims to present an overview of the research on cerebellar neurostimulation to modulate socio-affective functions in the healthy and clinical population. We decided to include in our search also studies employing NIBS in patients with other clinical pathologies, beyond SCA syndromes, in line with evidence reporting cerebellar structural and functional abnormalities in a large variety of neurological and neuropsychiatric conditions [76]. Indeed, the existence of cortico-cerebellar and cerebellar limbic networks involved in social cognition makes the cerebellum a promising target candidate to modulate socio-affective functions. Therefore, notwithstanding the differences in etiology-related factors, the effects on socio-affective functions achieved via cerebellar NIBS in other pathologies associated with cerebellar anomalies may generalize to the SCA population. Hence, this scoping review aims to foster a discussion on potential therapeutic approaches to treat the socio-affective symptoms afflicting SCA patients. We decided to perform a scoping review and not a systematic review due to the heterogeneity of the literature on the topic. This heterogeneity can be detected in the stimulation tools, in the outcome measures, in the clinical conditions as well as in the scopes. Indeed, the included studies aimed either at investigating the contribution of the cerebellum to specific socio-affective functions using neurostimulation tools or at examining the effectiveness of these tools targeting the cerebellum in reducing socio-affective symptoms or improving social functioning in clinical populations with cerebellar damage or with impairments in functions that involve the cerebellum. This review focused on articles published between January 2000 and December 2022 and included exclusively primary research studies. To our knowledge, no reviews on the same issue are available in the extant literature. Even though SCA syndromes represent a relatively rare condition with a low prevalence rate in the population, the costs and burdens associated with the disease, in all its clinical manifestations, have a significant impact on the quality of life of these patients and their caregivers [77]. Hence, reviewing the literature on this topic should be considered a matter of interest not only for research scopes but also for clinical practice.

## Materials and Methods

This review was conducted according to the framework proposed by Peters et al. [78] for scoping studies.

### Identifying the Review Questions

The research question was “*What is the evidence, described in the published literature, of cerebellar neuro-stimulation boosting effects on socio-affective functions in the clinical and healthy population?*”. The aims of the present review were to i) map and summarize the evidence on the boosting effects of cerebellar neuro-stimulation on socio-affective functions, and ii) develop a discussion on potential rehabilitation implications for hereditary ataxia patients.

### Inclusion Criteria

Criteria for study inclusion were the following: i) usage of NIBS techniques targeting the cerebellum in humans for ii) the modulation of socio-affective functions, as assessed by performance-based measures, questionnaires or qualitative outcomes (e.g., verbal report), reported in iii) research articles published in peer-reviewed English-language journals. Records considered as not pertinent were the following: animal studies, studies using deep brain stimulation techniques, or other stimulation tools (e.g., vagus nerve stimulation), applying NIBS targeting other brain areas or applying cerebellar NIBS but measuring exclusively non-social outcomes (i.e., other cognitive or motor functions or physiological measures), neuroimaging studies or studies applying any other investigation techniques but NIBS (e.g., pharmacological investigations), as well as any document other than primary research articles (e.g., review, book chapter, commentary etc.). At each selection process step, if one or more of these exclusion criteria were found in screening the record – thus, if information indicative of the presence of any exclusion criteria was detected in the title, abstract or text – then the record was not selected for inclusion. Studies applying NIBS in humans and examining both social and non-social outcomes were included, but only the information regarding the social outcomes was extracted and summarized in the following steps. We considered “socio-affective” a broad range of measures assessing social skills (e.g., actions comprehension or biological motion discrimination), mental health problems (e.g., depressive and/or anxiety symptoms severity), affective states or emotion regulation abilities.

### Search Strategy

The literature search was conducted by two authors, V.O and A.C., by screening scientific online databases (PubMed, Scopus and Web of Science) to identify pertinent studies, using the following text string: (“brain stimulation” OR neuromodulation OR “transcranial direct current stimulation” OR “transcranial magnetic stimulation” OR tES) AND (cerebellum OR cerebellar) AND (social OR emotion OR affective OR mood). The period considered for study inclusion was January 2000-December 2022. No other restrictions on document type or language of records were posed.

### Evidence Screening and Selection

**Fig. 1:**
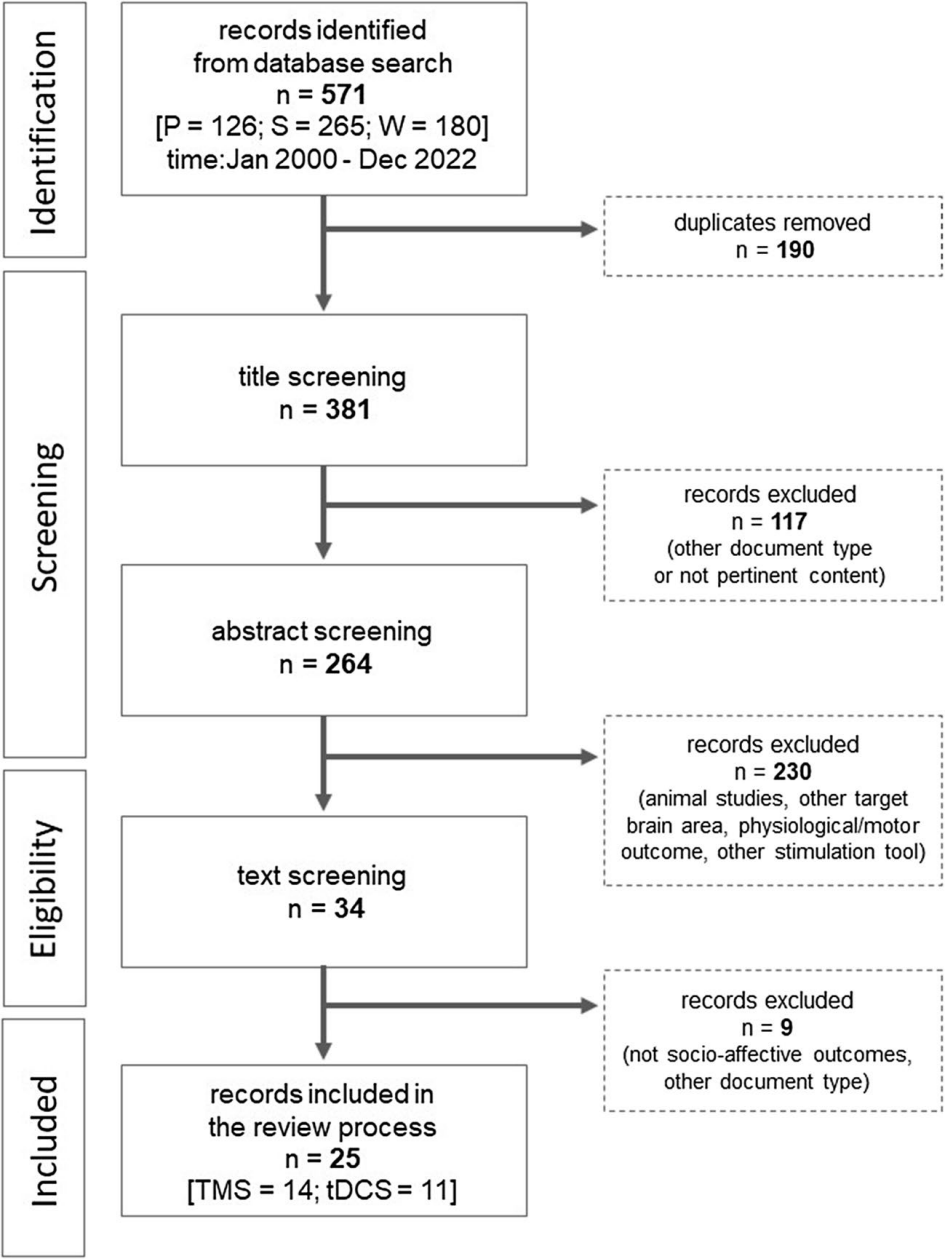
Flowchart of the study selection procedure

After the removal of duplicates, V.O. and A.C. screened the titles of the records, (title screening) identified by the search string and excluded those records not fitting with the topic of the review. Of the remaining records, they screened the abstract (abstract screening) and excluded those whose content was judged not to be relevant for the present review. In case some methodological information could not be retrieved by screening the abstracts, the two authors read the full texts (text screening) to determine whether to include the record. The list of records, after duplicates removal, was divided into two parts; one reviewer screened the first half, the other the second half. In case of disagreement in records selection for inclusion, other two authors, C.U. and Z.C., were consulted to discuss the reasons for the disagreement and to deliberate upon study inclusion. Figure 1 depicts the study selection procedure.

### Data Extraction and Charting

Data were charted referring to the review question “What is the evidence, described in the published literature, of cerebellar neuro-stimulation boosting effects on socio-affective functions in the clinical and healthy population?”.

The list of records selected for inclusion was divided into two parts. For each part, data were extracted by one reviewer and a 25% sample was checked for accuracy and completeness by the other [79].

Studies were categorized according to the neuro-stimulation technique applied, namely TMS or tDCS, and the target population, namely healthy volunteers or clinical samples. TMS and tDCS studies were schematized in separate tables, as the two techniques present different technical characteristics and modalities of usage. First, we reviewed TMS and then tDCS studies.

The table reporting the technical details of the TMS protocols (Table 1) summarizes the following information: authors and year of publication; type of stimulation protocol (e.g., repetitive or single-pulse); target site(s); type of coil used; frequency and intensity of the stimulation; number of sessions; timing of the stimulation (online or offline); whether or not an MRI-guided navigation system was used; information on whether TMS-induced sensations were reported or not. The table reporting the technical details of the tDCS protocols (Table 2) summarizes the following information: first author and year of publication; montage of electrodes; electrodes size; stimulation intensity; number of sessions; timing of the stimulation (online or offline); information on whether tDCS-induced sensations were reported or not.

**Table 1:** Technical details of the TMS protocols

	TMS								
	Author & year	Type	Target site(s)	Frequency & Intensity	Coil	N sessions	Timing	MRI-guided	TMS-induced sensations
*healthy volunteers*	Schutter et al., 2003 [80]	rTMS	Medial cerebellum Occipital cortex	25 Hz 80% MT	Iron-core	2	offline	no	not reported
	Schutter & van Honk, 2009 [81]	rTMS	Vermis Occiput	1 Hz 80% MT	Iron-core	3	offline	no	controlled
	Schutter et al., 2009 [82]	rTMS	Medial cerebellum Occipital cortex	20 Hz 80% MT	Iron-core	2	offline	no	controlled
	Demirtas-Tatlidede et al., 2011 [83]	iTBS	R, L & midline cerebellum	5 Hz 100% MT	Figure 8	3	offline	yes	reported
	Gamond et al., 2017 [84]	rTMS (triple-pulse)	R cerebellum dmPFC Early visual cortex	20 Hz 100% MT	Figure 8	1	online	MRI template	not reported
	Ferrari et al, 2018 [85]	rTMS (triple-pulse)	L paravermal cerebellum (Exp 1,2,3) Early visual cortex (Exp 1,2,3) Vertex (Exp 1)	20 Hz 100% MT	Figure 8	3 (Exp 1,2) 2 (Exp 3)	online	MRI template	not reported
	Ferrari et al., 2022a [53]	rTMS (triple-pulse)	L paravermal cerebellum Early visual cortex Vertex	20 Hz 100% MT	Figure 8	1	online	MRI template	not reported
	Heleven et al., 2021 [86]	rTMS	Vermis	1 Hz 80% MT (real) 10% MT (sham)	Double cone	1	offline	no	not reported
	Ferrari et al., 2022b [87]	Exp 1 & 3: triple-pulse Exp 2: single-pulse	L cerebellum Vermis Vertex	Exp 1 & 3: 20 Hz 100% MT	Figure 8	1	online	MRI template	reported
*patients*	Demirtas-Tatlidede et al., 2010 [88]	TBS	Vermis	5-Hz 100% MT	Figure 8	10	offline	yes	reported
	Tikka et al., 2015 [89]	iTBS	Vermis	5,6,7 Hz 100% MT	Double cone	10	offline	no	not reported
	Garg et al., 2016 [90]	rTMS	Vermis	5,6,7 Hz 100% MT	Double cone	10	offline	no	reported
	De Vidovich et al., 2016 [91]^	rTMS	L cerebellum	1 Hz 80% MT	Double butterfly	1	offline	no	controlled
	Brady et al., 2019 [92]	iTBS	Vermis	5 Hz 100% MT	Figure 8	10	offline	yes	not reported

^ The study of De Vidovich et al., [91] tested a group of healthy volunteers and a group of patients. However, as they applied the same stimulation protocol to both groups, the information is reported only once, in the patient section. In the TMS-induced sensation column, if a study did not describe any subjective impression or side effect but commented on the general tolerability or informed that information on tolerability was gathered by the experimenter, it was labeled as “controlled”. Abbreviations: rTMS, repetitive TMS,iTBS, intermittent Theta Burst Stimulation; R, right; L, left; dmPFC, dorsomedial prefrontal cortex; MT, Motor Threshold

**Table 2:** Technical details of the tDCS protocols

	tDCS							
	Author & year	Montage	Electrodes size	Intensity	Duration	N sessions	Timing	tDCS-induced sensations
*healthy volunteers*	Ferrucci et al., 2012 [54]	A: Midline 1 cm below the inion C: R deltoid	6 × 7 cm 6 × 7 cm	2 mA	20 min	1	offline	not reported
	Newstead et al., 2018 [93]	Exp1: A: L dlPFC C: R cerebellum Exp2: A: R cerebellum C: L dlPFC	5 × 5 cm 5 × 5 cm	2 mA	12 min	Single session group: 1 Repeated-sessions group: 3	offline	reported
	Gheorghe et al., 2021 [94]	A: Midline 1 cm below the inion C: R deltoid*	5 × 7 cm 5 × 7 cm	2 mA	15 min	1	online	reported
	Oldrati et al., 2021 [95]	A: Midline 2 cm below the inion C: R buccinator*	5 × 5 cm 5 × 5 cm	1.5 mA	20 min	3	online	reported
	Clausi et al., 2022 [96]	A: Midline 2 cm below the inion C: R deltoid*	5 × 7 cm 5 × 7 cm	2 mA	20 min	1	offline	reported
	Ma et al., 2023 [97]	A: Midline 2 cm below the inion C: R upper arm*	5 × 7 cm 5 × 7 cm	2 mA	20 min	1	online & offline	reported
*patients*	Ho et al., 2014 [98]	A: L supraorbital region C: Electrode's top edge centered over the inion	5 × 7 cm 5 × 10 cm	2 mA	20 min	20	offline	reported
	Benussi et al., 2021 [99]	A: Midline 2 cm below the inion C: Spinal lumbar enlargement (2 cm under T11)	7 × 5 cm 6 × 8 cm	2 mA	20 min	20	offline	reported
	D'urso et al., 2021 [100]	A: F3 C: R cerebellum (1 cm below and 4 cm lateral to the inion)	5 × 5 cm 5 × 5 cm	1 mA	20 min	20	offline	reported
	Maas et al., 2022 [101]	A: Midline 2 cm below the inion C: R deltoid	5 × 7 cm 5 × 7 cm	2 mA	20 min	10	offline	reported
	Ruggiero et al., 2022 [102]	A: Midline 1–2 cm below the inion C: R shoulder	5 × 7 cm 5 × 7 cm	2 mA	20 min	5	offline	not reported

^*^ The polarity of the electrodes was reversed, so that the cerebellum received both anodal and cathodal stimulation in distinct experimental conditions. Abbreviations: R, right; L, left; dlPFC, dorsolateral prefrontal cortex

The tables on the methodological aspects, for both TMS (Table 3) and tDCS (Table 4) studies, report this information: first author and year of publication; research design (specifying the within- and between-participants factors); whether the study was sham-controlled; applied a blinding procedure; conducted a power-analysis for the estimation of the sample size; reported the effect size(s); and included a follow-up to detect long-lasting effects of the stimulation; sample size and characteristics (health status or diagnosis, age, sex); whether or not medication intake and any (other) medical conditions of the participants were reported or controlled for; outcome measures and details of these (e.g., type of task); reference socio-affective domain (classified as “social” or “affective”). In this regard, the outcomes referring to mental health problems, symptoms severity, emotion regulation abilities, as well as changes in mood and affective states, were labeled as “affective”. The outcomes referring to the cognitive mechanisms involved in the processing of social stimuli (e.g., emotion or action processing) were labeled as “social”. Lastly, it was indicated whether each study reported a significant modulation effect on at least one socio-affective outcome from all those assessed.

**Table 3:** Methodological features of TMS studies

	TMS													
	Author & year	Study design (factors)	Sham-controlled	Blinding	Power analysis	Effect-size(s)	Follow-up	N; age; sex (F/M)	Diagnosis	Medication	Medical condition	Outcome measures	Outcome domain	Modulation effect
*healthy volunteers*	Schutter et al., 2003 [80]	within: target site, task condition	no (active control site)	single-blind	not reported	yes	no	5; 26–43; 5/0	NA	controlled	controlled	-Spontaneous verbal reports of mood changes	Affective	yes (B)
	Schutter & van Honk, 2009 [81]	within: target site	yes (sham coil)	single-blind	not reported	yes	no	12; 18–23; 12/0	NA	reported	controlled	-Emotion regulation -POMS	Affective	yes
	Schutter et al., 2009 [82]	within: target site	yes (sham coil)	double-blind	not reported	yes	no	15; M = 20.4; 15/0	NA	not reported	controlled	-Implicit emotion processing during color naming -PANAS	Social Affective	yes
	Demirtas-Tatlidede et al., 2011 [83]	within: target site	no (active control site)	not reported	not reported	no	no	12; M = 28.8; 6/6	NA	not reported	controlled	-Picture-evoked (IAPS) emotions rating -POMS -VAS for mood evaluation	Affective	yes (B)
	De Vidovich et al., 2016 [91]^	within: time	no	not reported	not reported	no	no	9; M = 31; 5/4	NA	controlled	controlled	-Affective Go/No-go	Social	no
	Gamond et al., 2017 [84]	within: target site, task condition	no (active control site)	single-blind	not reported	yes	no	20; M = 23.8; 10/10	NA	controlled	controlled	-Attitude priming	Social	yes
	Ferrari et al, 2018 [85]	within: target site	no (active control site)	not reported	not reported	no	no	76; M = 23.1; 56/20	NA	controlled	controlled	-Explicit emotion discrimination (Exp 1,2,3) -Implicit emotion processing during gender discrimination (Exp 1,2) -Control: gender discrimination of neutral faces (Exp 3)	Social	yes
	Ferrari et al., 2022a [53]	Exp1 within: target site, task condition Exp2 within: target site, task condition between: emotional valence	no (active control site)	single-blind	not reported	no	no	60; M = 23.7; 36/24	NA	controlled	controlled	-Emotion (body postures) discrimination	Social	yes
	Heleven et al., 2021 [86]	between: sham vs real	yes (low intensity)	not reported	yes	yes	no	46; M = 24.6; 32/14	NA	not reported	controlled	-Picture and Story sequencing of social scripts, true beliefs, and false beliefs -Control: sequencing of mechanical events	Social	yes (B)
	Ferrari et al., 2022b [87]	Exp1 within: target site Exp2 within: target site, task condition between: time Exp3 within: target site	no (active control site)	single-blind	not reported	yes	no	112; M = 23.5; 80/32	NA	controlled	controlled	-Biological motion discrimination	Social	yes
*patients*	Demirtas-Tatlidede et al., 2010 [88]	within: time	no	not reported	not reported	yes	yes	8; M = 41; 1/7	schizophrenia	reported	controlled	-PANSS -CDSS -Self-report POMS -CGI -VAS for mood evaluation	Affective	yes (B)
	Tikka et al., 2015 [89]	within: time	no	rater-blind	not reported	yes	no	11; M = 24.6; 3/8	schizophrenia	reported	controlled	-PANSS -CDSS	Affective	yes (B)
	Garg et al., 2016 [90]	between: sham vs real	yes (coil tilted of 45°)	double blind	not reported	yes	yes	40; M = 31.6; 7/33	schizophrenia	reported	controlled	-PANSS -CDSS	Affective	yes (B)
	De Vidovich et al., 2016 [91]^	within: time	no	not reported	not reported	no	no	8; M = 40; 4/4	borderline personality disorder	controlled	reported	Affective Go/No-go	Affective	yes (B)
	Brady et al., 2019 [92]	between: sham vs real	yes (shamming surface electrodes)	double-blind	not reported	no	yes	11; M = 35.6; 3/8	schizophrenia and schizoaffective disorder	reported	controlled	-PANSS	Affective	yes (B)

In the “Medication” and “Medical condition” columns, if a study did not report any information on medication intake or medical condition but stated that use of prescribed/illicit drugs and the health status of the participants were screened by means of a clinical interview or the administration of ad hoc-questionnaires, it was labelled as “controlled”. In the “Modulation effect” column, if a study reported a significant boosting (i.e., positive) effect on at least one socio-affective outcome among all those assessed, it was labeled as “yes (B)”, where “B” indicates the boosting nature of the effect. If a study reported a significant negative, impairing effect, it was labeled as “yes”

Abbreviations: NA, not applicable; POMS, Profile of Mood States; PANAS, Positive and Negative Affect Schedule; IAPS, International Affective Picture System; VAS, visual analogue scale; PANSS, Positive and Negative Syndrome Scale; CDSS, Calgary Depression Scale for Schizophrenia; CGI, Clinical Global Impressions scale

^ Information extracted from De Vidovich et al., [91] on the healthy control group is reported in the upper part of the table (healthy volunteers section), while information on the group of patients is reported in the lower part (patients section)

**Table 4:** Methodological features of tDCS studies

	tDCS													
	Author & year	Study design (factors)	Sham-controlled	Blinding	Power analysis	Effect-size(s)	Follow-up	N; age; sex (F/M)	Diagnosis	Medication	Medical condition	Outcome measures	Outcome domain	Modulation effect
*healthy volunteers*	Ferrucci et al., 2012 [54]	within: polarity, time, task condition	yes	single-blind	not reported	no	no	21; 20–49; 12/9	NA	controlled	controlled	-Facial Emotion Recognition -VAS for mood evaluation	-Social -Affective	yes (B)
	Newstead et al., 2018 [93]	Exp 1 between: polarity within: time Exp 2 within: time	yes (Exp 1) no (Exp 2)	single-blind	not reported	yes	no	Exp 1 (single session) 44; M = 21.9; 30/14 Exp 1 (repeated sessions) 21; M = 21.4; 11/10 Exp 2 (single session) 23; M = 20; 16/7 Exp 2 (repeated sessions) 11; M = 23.3; 8/3	NA	not reported	controlled	-POMS -VAS for mood evaluation	Affective	yes (B)
	Gheorghe et al., 2021 [94]	between: polarity within: time	yes	single-blind	not reported	yes	no	45; M = 22.0; 26/19	NA	controlled	controlled	-POMS -VAS for mood evaluation	Affective	no
	Oldrati et al., 2021 [95]	within: polarity, time, task type	yes	single-blind	yes	yes	no	24; M = 22.5; 18/6	NA	not reported	controlled	-Social actions prediction -Control: non-social events prediction	Social	yes (B)
	Clausi et al., 2022 [96]	between: polarity within: time	yes	double-blind	no	no	no	48; M = 25.7; 26/22	NA	controlled	controlled	-Digital RMET -VAS for anxiety and fatigue evaluation	-Social -Affective	yes (B)
	Ma et al., 2023 [97]	between: polarity, task type within: time, task condition	yes	single-blind	yes	yes	yes	106; M = 20.3; 81/25	NA	not reported	not reported	-Implicit belief SRT -Control: Implicit cognitive SRT	Social	no
*patients*	Ho et al., 2014 [98]	between: montage (fronto-occipital vs fronto-cerebellar) within: time	no	not reported	not reported	no	yes	14; M = 44.9; 6/8^	major depressive disorder	reported	controlled	-MADRS	Affective	yes (B)
	Benussi et al., 2021 [99]	sham controlled phase between: stimulation (sham vs real) within: time open-label phase within: time	yes	double-blind	yes	yes	yes	61; M = 56.9; 34/27	neuro-degenerative ataxia	controlled	controlled	-CCASS -SF-36	-Social -Affective	no
	D'urso et al., 2021 [100]	within: time	no	not reported	not reported	no	no	7; M = 11.0; 1/6	autism spectrum disorder	reported	reported	-ABC -VAS for symptoms severity evaluation	-Social -Affective	yes (B)
	Maas et al., 2022 [101]	between: stimulation (sham vs real)	yes	double-blind	yes	no	yes	20; M = 51.9; 8/12	spino-cerebellar ataxia type 3	reported	controlled	-PHQ-9 -POMS	Affective	no
	Ruggiero et al., 2022 [102]	within: stimulation (sham vs real), time	yes	double-blind	not reported	no	no	9; 42–77; 4/5	idiopathic parkinson disease	reported	controlled	-Facial Emotion Recognition -VAS for mood evaluation	-Social -Affective	yes (B)

In the “Medication” and “Medical condition” columns, if a study did not report any information on medication intake or medical condition but stated that use of prescribed/illicit drugs and the health status of the participants were screened by means of a clinical interview or the administration of ad hoc-questionnaires, it was labelled as “controlled”. In the “Modulation effect” column, if a study reported a significant boosting (i.e., positive) effect on at least one socio-affective outcome among all those assessed, it was labeled as “yes (B)”, where “B” indicates the boosting nature of the effect. If a study reported a significant negative, impairing effect, it was labeled as “yes”. Abbreviations: NA, not applicable; VAS, visual analogue scale; POMS, Profile of Mood States; RMET, Reading the Mind in the Eyes Test; SRT, Serial Reaction Time; MADRS, Montgomery Asberg Depression Rating Scale; CCASS, Cerebellar Cognitive Affective Syndrome Scale; SF-36, Short Form Health Survey 36; ABC, Aberrant Behavior Checklist; PHQ-9, Patient Health Questionnaire-9. ^Only information on the sub-sample undergoing the fronto-cerebellar stimulation is reported

### Analysis of the Evidence and Presentation of Results

The first section describes the results of the research strategy and selection process. In the second section, data were synthesized in a descriptive format, in separate paragraphs and tables according to the specific neuro-stimulation tool applied in each study, to identify different aspects of the literature as outlined in the key question. The occurrence of stimulation technical details, methodological choices and outcome characteristics were reported.

## Results

### Study Selection for Review

The literature search identified a total of 571 records, 126 from Pubmed, 265 from Scopus and 180 from Web of Science. After the removal of duplicates (n = 190), the titles of 381 records were screened. Of these, 117 records were excluded as non-pertinent whenever the title was indicative of the presence of one or more of the exclusion criteria described above. The abstracts of the remaining records (n = 264) were screened against the inclusion/exclusion criteria. This stage of the selection process led to the identification of 34 potentially eligible studies. After reading the text of these records, 9 were further excluded. Thus, 25 records were considered eligible and included in data extraction and charting.

### TMS Protocols

Out of the 25 records included in the data extraction and charting phase, 14 (56%) applied the TMS. Of these, 9 (64.3% of the TMS studies) tested healthy participants, whereas 5 (35.7%) enrolled different clinical populations. Three studies (21.4%) applied a low frequency (1 Hz) repeated TMS protocol, 5 applied a theta-burst stimulation protocol, with frequencies ranging from 5 to 7 Hz, and the remaining 6 studies (42.9%) opted for a high frequency protocol, at 20 or 25 Hz. In one of the latter studies, the authors included an experiment delivering single-pulse TMS. The medial portion of the cerebellum was chosen as the main target site in 10 studies (71.4%). In 2 of these, TMS was also delivered to the left cerebellar hemisphere, and one of the 2 also targeted the right cerebellar hemisphere. Of the remaining studies, 3 targeted only the left cerebellar hemisphere and only one the right cerebellar hemisphere, besides other target non-cerebellar areas. In studies stimulating other cerebral areas (50%), the occipital cortex was chosen as a control site in 6 cases—given its proximity to the cerebellum and to rule out the possibility that any observed effect may depend on indirect stimulation of the visual cortex [103]—and the vertex in 3 cases.

As for the intensity parameter, 5 studies (35.7%) stimulated at 80% of the participants’ motor threshold (MT), 9 at 100%.

For what concerns the coil type and geometry, half of the studies used a figure-of-eight coil—likely stimulating more superficial, posterior regions of the cerebellum—4 studies used a double cone coil—recommended for effective cerebellar stimulation [104]—and 3 an iron-core coil.

As emerged for tDCS, clinical studies included a greater number of TMS sessions (n = 10) than studies on healthy volunteers. Only in De Vidovich et al. [91], which consists of a one-shot experiment, the clinical sample underwent a single TMS session. In 4 studies, all applying a triple-pulse paradigm, the TMS was delivered online, thus during stimuli presentation, whereas the remaining 10 studies adopted an offline paradigm. Although neuronavigated-TMS on individual magnetic resonance images (MRI) scans is encouraged to enable precise targeting and decrease interindividual variability [105], only 3 studies complied with this requirement. Other 4 studies, conducted by the same research group, localized the target areas employing stereotaxic navigation on individualized MRI scans, which were obtained through a 3D warping procedure fitting a high-resolution MRI template with the participant's scalp model and craniometric points. In the remaining 7 studies, the coil positioning was based on anatomical landmarks. In contrast with tDCS studies, half of the TMS studies did not report any information on cerebellar TMS tolerability and correlated sensations, including but not limited to pain and discomfort due to the contraction of neck muscles, often associated with this type of stimulation. Only 4 studies reported some degree of information on minor side effects (e.g., headache or sleepiness). Three studies did not describe any side effect or subjective sensation but commented that the stimulation was well tolerated or that information on tolerability was gathered by the experimenter.

### TMS Studies: Methods and Characteristics

This paragraph provides a descriptive overview of the methodological features—schematized in Table 3—of the TMS studies.

Out of the 14 TMS studies, only 3 (21.4%) studies adopted a between-participant design, in which participants received either real or sham stimulation. The remaining 11 (78.6%) studies adopted a within-participant design, with all the participants receiving real TMS over the cerebellum, selected as the main target area, and a sham stimulation (in 2 cases) or real stimulation in other control target areas (in 6 cases). Three studies did not include any control condition (nor sham or active control sites). However, among these, the study by De Vidovich and colleagues (2016) aimed at comparing the performance of a group of patients with the performance of a group of healthy controls.

Overall, 5 (35.7%) studies were sham-controlled. The sham condition was obtained with either one of the following methods: using a modified coil, able to mimicking the sound click and sensation of real TMS, in which a metal plate was built in the housing directly under the iron-core (in 2 cases); setting the stimulation to a low intensity (10% of the individual MT); tilting the coil of 45 degrees; or applying shamming surface electrodes at the participants’ neckline, to simulate the tactile effects of the stimulation.

As for the blinding procedure, less than half of the studies (28.6%) adopted a double-blind procedure, while 5 studies adopted a single-blind procedure. No indication of the use of any blinding method was found in the remaining 5 studies. Surprisingly, only one study reported to have conducted a power analysis for the estimation of the sample size. In all other studies, no evidence of this analysis was found. For what concerns the inclusion of follow-up measures, they were detected only in 3 studies, all examining cerebellar TMS potential therapeutic effects in patients.

In total, the TMS studies included in the review process tested 445 participants, of which 367 healthy volunteers and 78 patients. All participants were adults. Except for the study of De Vidovich and colleagues (2016), which consisted of a one-shot experiment comparing the performance in a task of a group of patients with borderline personality disorder with that of a group of healthy controls, all 4 clinical studies enrolled patients diagnosed with schizophrenia and schizoaffective disorder. Cerebellar abnormalities have been observed in both borderline personality disorder and schizophrenia [106, 107].

The use of medications by the patients was either reported or controlled for in all clinical studies, including the sub-sample of patients tested by De Vidovich et al., [91]. For what concerns the studies testing healthy volunteers, only 3 studies failed to report this information. Evidence of screening of the participants’ health status was found in all studies. For what concerns the outcome measures, the review process depicts a heterogeneous picture of socio-affective functions, types of tasks and paradigms. The measures whose domain was labeled as “social” were all performance-based and included: explicit emotion processing tasks from facial expressions or body postures; implicit emotion processing tasks (e.g., during a color naming task or a gender recognition task); a Go/No Go task using words of either positive or negative valence; an attitude priming task requiring to categorize the valence of a series of adjectives primed by either an in-group or an out-group face; a picture and story sequencing task requiring to order sequences of actions involving true and false belief stories; and a biological emotion discrimination task. All these tasks were administered to healthy volunteers. Only 4 studies on healthy volunteers aimed at evaluating changes in mood and affective states. More precisely, in the study by Schutter and colleagues (2003) the elevation in mood observed after cerebellar TMS was not an outcome measure the authors had planned to monitor, but rather it was spontaneously reported by all the participants [109]. Affective measures administered to healthy volunteers included: an emotion regulation task and an emotion rating task following the exposition to emotion-eliciting pictures; standardized questionnaires for the evaluation of mood; VAS for the evaluation of changes in mood and affective states. All clinical studies assessed affective outcomes, including measures of affective states and symptoms severity, favoring standardized questionnaires.

With regards to the findings, 8 (57.1%) studies, including all the clinical studies, found that cerebellar TMS significantly improved at least one of the socio-affective outcomes among the assessed ones. It has to be noted that all the studies testing healthy volunteers reported a significant modulation effect of the stimulation, although in 6 cases (42.9%) these effects were not found to boost the performance but provided evidence on the involvement of the cerebellum in the socio-affective function examined. Only the one-shot experiment conducted by De Vidovich and colleagues (2016) did not find any significant effect in the healthy control group, whereas a positive effect of the stimulation was found in the group of patients. This could pave the way for a discussion of publication bias. The only study on healthy participants in which no effects were reported was that of De Vidovich et al. [91], but these negative results were published because matched with positive findings in patients. One may suspect that other studies with all negative findings could not be published because they are less likely to be accepted by journals [108]. That said, with the exception of the study by De Vidovich et al. [91], significant cerebellar stimulation effects on socio-affective functions were found in all the TMS studies included in the review process.

### tDCS Protocols

Out of the 25 records included in the data extraction and charting phase, 11 (44%) were tDCS studies. Of these, 6 (54.5% of the tDCS studies) tested healthy participants, whereas 5 (45.5%) enrolled different clinical populations. The majority of the studies (81.8%) targeted the medial cerebellum, while only 2 targeted the right cerebellar hemisphere. Excluding intra-cephalic montages, the right deltoid muscle was chosen as the reference region in 6 studies. Only one study applied the reference electrode over the right buccinator, and only one study targeted the spinal lumbar enlargement. In the healthy sample studies, the cerebellum was targeted by both the anode and cathode electrodes in a between-participants design (4 studies), where participants received either anodal or cathodal cerebellar stimulation, or in a within-participant design (2 studies), where all participants underwent both stimulation types in distinct time points. The cerebellum received active anodal stimulation in 3 studies and active cathodal stimulation in the remaining 2 studies on patients. The intensity was set at 2 mA in the majority of the studies (81.8%), while in one case it was set at 1.5 mA. The only study on children applied an intensity of 1 mA, in accordance with the recommendations on the safety and tolerability of tDCS in the pediatric population [109]. The stimulation lasted 20 min in 9 studies and in no case it exceeded this amount of time, remaining within the recommended limits of safety [110]. It is not surprising that clinical studies involved a greater number of stimulation sessions, ranging from 5 sessions (in one study) to 20 sessions (in 3 studies), likely to maximize the potentially beneficial effects of the stimulation over time. As for the timing, only 3 studies (27.3%) examined the effect of the stimulation on the outcome online, namely during the delivery of the current, whereas in all other cases the effects were assessed at the end of the stimulation (offline). In conformity with the guidelines [110], 81.8% of the studies reported that information on tDCS tolerability was gathered from participants. Yet, only four studies described the specific tDCS-induced sensations (Gheorge et al., 2021,[93, 98]. Tingling, skin redness and trouble in concentrating (or dizziness) were reported in all three studies,itching, burning sensation, headache and sleepiness were reported in two of the studies; scalp pain was reported only in one study, as were nausea, fatigue and mood change. One study compared the extent of all the above-mentioned sensations during real and sham stimulation conditions values were very low for all measures and they were comparable between the two stimulation conditions, except that itching and skin redness were rated as greater for real (anodal) than sham stimulation [97]. Finally, only in 2 studies no information on tolerability was detected during data extraction.

### tDCS Studies: Methods and Characteristics

This paragraph provides a descriptive overview of the methodological features of the tDCS studies, schematized in Table 4.

Out of the 11 tDCS studies, 6 (54.5%) investigated polarity-dependent effects (i.e., anodal vs. cathodal stimulation); in 2 cases in a within-participants design and in 4 cases in a between-participants design. None of these were clinical studies, in which either anodal or cathodal cerebellar effects were investigated. The majority of the studies (81.8%) were sham-controlled. Newstead and colleagues [93] conducted a sham-controlled experiment (Exp 1) and an experiment (Exp 2) in which tDCS effects were examined over time with no control condition. Of those studies (18.2%) not involving a sham-control condition, one compared a fronto-occipital montage to a fronto-cerebellar montage and the other one simply examined tDCS effects over time with no control condition. Less than half of the studies (36.4%) adopted a double-blind procedure, even though its adoption is recommended to minimize the potential effects of research bias when collecting data [111]. Five studies (45.5%) adopted a single-blind procedure, in which only participants, but not experimenters, were blind to the type of stimulation delivered. Only in 2 studies no information on the blinding procedure was detected during data extraction. As for the power analysis calculation for estimating the sample size, a benefit of conducting this analysis is that it helps researchers to maximize the probability of observing the expected (significant) effect in the smallest sample size suitable for the purpose [112]. However, only 4 studies reported a power analysis for sample size estimation. In one further case, the sample size was estimated based on the sample size reported in similar research. In 6 cases, no information on power analysis was reported.

There is consensus that authors should report not only indexes of statistical significance, examining whether the findings are likely to be due to chance, but also effect sizes, which help the reader to understand the magnitude of the observed differences found between conditions (or groups, treatments etc.) [113]. Nevertheless, only 5 studies complied with this recommendation. For what concerns the inclusion of follow-up measures, 4 studies, of which 3 on patients, monitored potential long-lasting effects of the stimulation after the last stimulation session. Five studies on healthy volunteers and 2 on patients consisted of one-shot experiments and, thus, did not plan any follow-up phase.

In total, the tDCS studies included in the review process tested 422 participants, of which 311 healthy volunteers and 111 patients. Only one study [100] recruited children. Among the clinical studies, 2 aimed at examining potential therapeutic effects of cerebellar tDCS on patients diagnosed with neurodegenerative ataxia of different etiology. The other 3 studies focused on the following pathologies of the nervous system: major depressive disorder, autism spectrum disorder (in children) and idiopathic Parkinson's disease. All these disorders have been reported to display structural and functional anomalies of the cerebellum [114–116].

For both clinical and healthy sample studies, information was reported on whether medication intake and any medical condition of the participants (besides the main diagnosis for which patients were enrolled in the first place) were reported or controlled for. Only 3 (27.3%) studies, all on healthy participants, did not report any information on medication intake, while the remaining 8 studies either reported which medications the participants were taking at the time of the study or stated that participants were screened for the use of medications. Only in one study, no evidence of screening of the participants’ health status was found, whereas in all the other studies (90.9%) the presence of any medical conditions was either reported or controlled for.

Similarly to what was observed reviewing the TMS studies, tDCS studies presented a high heterogeneity in the socio-affective outcomes, type of tasks and paradigms. Most studies (81.8%) measured exclusively (in 4 cases) or also (in 5 cases) “affective” outcome, where this term refers to measures assessing mental health problems, symptoms severity, emotion regulation abilities or changes in mood and affective states. A visual analog scale (VAS) was used to evaluate changes in mood and affective states in 6 studies. Four out of 5 studies examining patients used standardized questionnaires for the evaluation of symptom severity and psychological adjustment. The measures labeled as “social” were all performance-based and included measures of the ability to recognize emotion from pictures of faces (facial emotion recognition task) or of the ability to infer mental states from pictures of eyes (Read the Mind in the Eye Test, RMET). Another task required participants to form predictions of social actions, then tested in conditions of perceptual uncertainty, based on the probability of co-occurrence between a particular action and contextual elements (social actions prediction task). Lastly, it was labeled “social” a task that required participants to learn a sequence that included information about others’ beliefs, which might converge with or differ from reality, resulting in true and false beliefs respectively (Implicit Belief Serial Reaction Time task).

Overall, 7 (63.6%) studies found that cerebellar tDCS significantly improved at least one of the socio-affective outcomes among all those assessed. On the other hand, 4 studies did not find any significant effect (Table 4). Among these, Benussi and colleagues (2021) reported a null effect of the stimulation on the Cerebellar Cognitive Affective Syndrome Scale (CCAS) total score, which provides a global indication of both the cognitive and affective skills of the patient. However, they did not differentiate the affective from the cognitive sub-score. Hence, no definitive conclusion on the potential effect of cerebellar tDCS on the affective components as measured by this scale could be drawn in the context of this study.

## Discussion

The cerebellum plays a crucial role in socio-affective functions [1, 2], likely mediating predictive mechanisms through the generation and learning of social action sequences [12]. Indeed, patients suffering from cerebellar alterations, such as hereditary ataxia, often show difficulties in recognizing and inferring others’ mental states [13, 39, 45, 47] and in regulating their own emotional state. Nevertheless, the available rehabilitation protocols for these conditions mainly focus on sensory-motor symptoms, while paying little attention to socio-affective deficits. Among non-pharmacological approaches, treatments applying NIBS (alone or in combination with other conventional interventions) seem to be effective for rehabilitation purposes on cognitive and socio-affective functions in several clinical conditions (e.g., [117, 118]. Here, we identified and summarized the evidence on the boosting effects of cerebellar neurostimulation on socio-affective functions to discuss potential rehabilitative implications for hereditary ataxia patients.

### Cerebellar Neurostimulation Boosting Socio-Affective Functions in Healthy Individuals

In most of the reviewed studies, the stimulation of the posterior cerebellum (with both TMS and tDCS) was effective in modulating healthy participants’ abilities in processing others’ mental states, from low-level motor intentions to emotions and higher-level mental states (e.g., beliefs and attitudes). In particular, tDCS applied over the medial cerebellum improved the recognition of others’ emotional states as expressed by facial expressions [54] and pictures of the eye region [96]. Furthermore, high-frequency rTMS over the same region enhanced emotional facial expressions recognition, even when emotional expressions were irrelevant to the task at play (i.e., implicit) [82]. Similarly, triple-pulse TMS over the paravermal cerebellum (in particular Crus I/Crus II) affected the explicit and implicit processing of happy and angry facial expressions [85]. Important indications for the possible explanation of why cerebellar stimulation improves emotion recognition come from empirical evidence suggesting that the posterior cerebellum may be selectively involved in the processing of negative emotional signals. Indeed, Ferrucci and colleagues [54] observed a selective improvement in the processing of negative emotional facial expressions following both anodal and cathodal tDCS over the medial cerebellum. Similarly, triple-pulse TMS over the paravermal cerebellum affected the discrimination of negative body expressions (i.e., anger or sadness), leaving the discrimination of positive body expressions (i.e., happiness and surprise) unchanged [53]. These findings support prior neuroimaging evidence reporting selective cerebellar activations in response to negative emotional cues [119, 120]. The selective valence-related role of the posterior cerebellum may depend on its role in predictive mechanisms. Within this framework, an agent expressing a negative emotion (e.g., anger or fear) may signal a potential danger to the perceiver and trigger (motor) “fight or flight” reactions (e.g., [121]. Cerebellar neurostimulation may thus potentiate the preparatory mechanisms implemented by the cerebellum that may help to respond to a potential threat.

Direct evidence on the role of the cerebellum in predictive mechanisms in social cognition comes from the study by Oldrati et al. [95] in which participants had to predict an agent’s action intention based on available contextual information. Anodal tDCS over the medial cerebellum improved the ability to infer and predict others’ action intentions when these were embedded in moderately informative contexts, while cathodal stimulation hindered participants’ sensitivity in predicting actions only when presented in strongly (but not moderately) informative contexts. Critically, tDCS did not affect a non-social control task requiring participants to predict the movements of physical shapes. This finding seems to suggest a specific and beneficial effect of cerebellar stimulation (at least of its medial part) in forming expectations related to social events. In particular, the cerebellum would play a crucial role in context-based prediction, where the available context (e.g., a particular place/situation/person or objects available in a scene) activates stored mental models of what can be expected in similar contexts [122]. This allows the prediction of others’ actions, emotions, or mental states and the control of ongoing inter-actions necessary for successful social interactions. Further support for the role of the cerebellum as a predictive device acting based on contextual information comes from a TMS study testing the neural correlates of stereotypical associations, which are implicit social associations that are prevalent in a specific social context/culture [84]. In this study, triple-pulse TMS over the (right) posterior cerebellum between the presentation of an in-group or out-group face and a trait adjective that participants had to evaluate, affected the stereotypical association between positive traits and in-group members, thus suggesting that the posterior cerebellum processes social signals depending on the associated/learned social context. Within the predictive framework, the role of the cerebellum would be to identify and predict sequences of a person’s (social) actions by supporting the explicit or implicit learning of frequently executed sequences of actions and mental states [14, 123, 124]. Accordingly, Heleven et al. [86] showed that low-frequency rTMS over the medial cerebellum significantly improved healthy participants’ performance in a Picture and Story sequencing task, which involved the explicit generation of the correct chronological sequence of social and non-social stories. Crucially, no difference was observed between false belief and mechanical (non-social) control stories, suggesting a cerebellar domain-general role in sequence generation. Similarly, anodal tDCS over the medial cerebellum improved the ability to implicitly learn non-social sequences [97].

As for affect regulation, the reviewed studies provide interesting data, though with some less consistent findings. Following 20 min of high-frequency rTMS over the medial cerebellum, participants spontaneously reported elevations in alertness and elevated mood [80], whereas low-frequency TMS impaired participants’ emotion regulation abilities as measured through self-compiled scales [81]. Mood elevation has been also observed following the simultaneous stimulation of the right cerebellar hemisphere and left dorsolateral prefrontal cortex, with accumulative and potentiated effects following successive stimulations [93]. However, it is difficult to disentangle the selective effect of cerebellar stimulation from the well-established effects of stimulation of the left prefrontal cortex on mood regulation [125]. Affect regulation might be seen as part of a body energy regulation process that aims to maintain (body) energy balance (i.e., homeostasis) by predicting the body’s needs and preparing to meet them [126]. Thus, the role of the cerebellum in predictive mechanisms might explain also its involvement in the regulation of one’s affective states. Nevertheless, it is important to note that several studies did not observe any beneficial effect on mood or affect regulation following either anodal or cathodal tDCS [54, 94, 96], high-frequency TMS [82] or iTBS [83] targeting the cerebellum, an issue that requires further investigation.

### Cerebellar Neurostimulation Boosting Socio-Affective Functions in Clinical Populations

Among the reviewed articles, only two applied cerebellar NIBS on hereditary ataxia patients in randomized, double-blind, sham-controlled trials [99, 101]. In Benussi et al., [99], repeated sessions of anodal tDCS using a cerebello-spinal montage (i.e., one electrode over the medial cerebellum and the other over the spinal lumbar enlargement) improved neurodegenerative ataxia patients’ motor abilities, cognition, and quality of life for weeks after the treatment with additive effects after two repeated treatments. Note that socio-affective functions were not addressed with specific outcome measures in this study, but they were measured only as part of broader scales or questionnaires assessing also cognitive abilities or more general quality of life aspects. Maas et al. [101], though, observed no effect on motor, cognitive and patient-reported outcomes evaluating depressive symptoms and mood states in a cohort of patients with a specific type of hereditary ataxia, SCA3 (not included in [99]) following repeated sessions of anodal stimulation over the medial cerebellum (with reference electrode over the right deltoid muscle). Hence, given the differences in patients’ diagnosis, electrode montages, and outcome measures, it is premature to draw conclusions about the effectiveness of cerebellar neurostimulation in enhancing hereditary ataxia patients' socio-affective functions based on this evidence and further systematic investigations are necessary.

Nevertheless, studies employing cerebellar stimulation in other neurological and psychiatric conditions that are associated with cerebellar alterations may offer promising insight towards the implementation of innovative treatment protocols for hereditary ataxia patients also in the socio-affective domain. For instance, the ability to make accurate predictions in both social and non-social domains is impacted in autism spectrum disorder (ASD) patients [127, 128], who also show impaired ability to make predictions about their internal state [129], likely due to cerebellar structural and functional alterations (for review see [130, 131]). Among the reviewed studies, only one targeted the cerebellum in a small cohort of children with ASD [100], and showed a general reduction in symptoms global severity, particularly those related to social withdrawal and lethargy, hyperactivity, and mood, following 20 daily sessions of cathodal stimulation of the right cerebellar hemisphere and anodal over the left dorsolateral prefrontal cortex. Several studies among those considered here applied cerebellar NIBS to boost socio-affective functions in schizophrenia, a psychiatric condition in which imprecise predictive coding may represent a core pathological factor [106, 132], with symptoms’ severity being associated with reduced cerebellar grey matter volume and altered resting-state functional connectivity [133–135]. In line with this, a reduction of negative symptoms, including blunted affect, decreased motivation, social withdrawal, and anhedonia (as assessed by clinicians) has been observed following repeated sessions of iTBS over the medial cerebellum [88–90, 92]. In addition, patients reported positive effects on mood and depressive symptoms [88–90], but see [92]. Furthermore, patients with mood disorders may also present cerebellar gray matter loss and altered cerebellar-prefrontal connectivity [3, 114, 136]. Crucially, these difficulties in emotion regulation have also been associated with inefficient predictive coding [137] that leads to uncertainty and chronically elevated levels of distress and negative mood [126]. Accordingly, four weeks of simultaneous cathodal cerebellar stimulation and anodal left prefrontal cortex stimulation resulted in beneficial modulations of mood and depressive symptoms in patients with Major Depression, although with a weaker effect compared to a fronto-occipital montage [98].

Interestingly, cerebellar neurostimulation has been proven to be effective in modulating social and affective functions of patients with neurological and psychiatric conditions that might not be directly associated with cerebellar dysfunctions [91, 102]. Five consecutive days of anodal tDCS over the medial cerebellum, while not affecting patients’ mood evaluations, enhanced the recognition of specific negative emotional states in patients with Parkinson’s disease [102], in line with brain stimulation studies on healthy individuals [53, 54]. This effect may depend on cerebellar tDCS affecting a widespread network that enhances the processing of emotionally salient stimuli, as those with negative valence, Similarly, the positive effects on impulse control observed in borderline personality disorder patients following low-frequency rTMS over the left cerebellar hemisphere might be due to the stimulation exerting a facilitating effect on behavioral control mechanisms tapping on prefrontal regions through the modulation of cerebello-prefrontal connections [91].

### Considerations and Recommendations

The reviewed studies present high heterogeneity in their methodology, in terms of different experimental designs, outcome measures, cerebellar target sites as well as stimulation parameters, including coil type (for TMS), and electrode montage and size (for tDCS). On one hand, this discrepancy may explain some inconsistency in the overall results, on the other hand, it complicates the identification of the stimulation protocols that may be more effective in boosting socio-affective functions. Nevertheless, despite this variability and the scarce literature on hereditary ataxia patients on this topic, the present review provides encouraging perspectives on the possibility of using cerebellar neurostimulation to improve the ability to process others’ mental states in healthy individuals and reduce social and affective symptoms in some neurological and psychiatric populations with cerebellar damage or with impairments in functions that involve the cerebellum. Yet, we cannot exclude that the presence of publication bias may have influenced our conclusions, leading to an overestimation of the benefits of NIBS due to a reduced tendency to disseminate null results. Therefore, the interpretations provided here must be taken with caution while awaiting both correctly powered and replication studies. Another important consideration is that, although defining a time window is mandatory when conducting a systematic review to allow the reproducibility of results, it also means missing to include relevant, recently published studies that could be beneficial for the review (see for instance [138–140]).

Furthermore, most of the reviewed studies targeted medial cerebellar regions, aiming at Crus I/II, in line with neuroimaging evidence reporting the functional connections between these sectors and the salience network [26, 27, 141], dedicated to the detection and attentional orientation towards emotional/salient stimuli [142], to select the more appropriate emotional response based on the individual’s current state (for a review see [143]). Only few articles targeted lateral sectors, in both the left [53, 83, 85, 87, 91] and right hemispheres [83, 84, 93, 100]. However, whereas low-level social operations, including the processing of others’ emotional expressions and one’s own affect regulation, may recruit medial regions [144], more complex social functions, including those involved in the processing of social sequences to predict higher-level mental states, such as beliefs, may be localized in slightly more lateral sectors [145], see also [139]). Accordingly, TMS “virtual lesion” studies showed that more lateral hemispheric regions are causally involved in processing others’ action intention [87], and others’ emotional and mental states inferences [53, 84, 85]. In light of the potential functional distinctionof social-affective operations along the medial–lateral axis of the cerebellum [144, 145], future studies are needed to test the effect of lateral cerebellar stimulation in boosting socio-affecting functions, which may be particularly effective in reducing patients’ difficulties with more complex and high-level social inferences. On this point, it is worth noting that among the reviewed articles, only 7 used individual MRI [83, 88, 92] or estimated-MRI [53, 84, 85, 87], to localize the target regions, in all other cases, the regions of interest were localized by using craniometrics points, which provide a less precise localization. Moreover, in the tDCS studies, the stimulation was applied through two relatively large electrodes (i.e., at least 25 cm2) that, although effective in modulating cerebellar activity, have low spatial resolution with stimulation spreading to at least part of the hemispheres [146, 147]. Hence, while the neurostimulation of the medial cerebellum seems promising in boosting socio-affective functions, it is not possible to draw sufficient conclusions regarding its specificity and the potential role of hemispheric regions. There is a need for research applying more precise localization methods such as individual MRI, computational modeling of the electric field distribution and, for tDCS studies, appropriate montage solutions using either smaller electrodes [148] or High-Definition tDCS (e.g., [149]) to improve stimulation focality. If this issue is relevant when targeting all cerebral regions, it is even more important for cerebellar stimulation, considering the convoluted structure of the cerebellar cortex, and particularly in conditions of increased atrophy or sulci width alterations, as in the case of hereditary ataxia. Moreover, future studies should evaluate the effects of the modulation of distinct cerebellar sectors through a comprehensive set of socio-affective outcome measures. Specifically to obtain a clearer view of the best cerebellar sector to target to potentiate distinct socio-affective functions, affect regulation abilities should be assessed using both questionnaire and experimental performance tasks, whereas tasks varying for complexity and abstraction of the required inferences should be employed to systematically evaluate the ability to process others’ mental states. Another possibility that future studies may address is the application of frequency-tuned stimulation for the treatment of diseases manifesting with abnormal cerebellar oscillatory activity. This approach, for which both TMS and specific types of transcranial electrical stimulation (such as transcranial alternating current stimulation, tACS) can be used, consists of “entraining” specific frequencies in the endogenous brain oscillatory activity that is associated with a specific function (see [150, 151]) and it is effective in modulating cerebellar excitability in a time- and frequency-dependent manner (e.g., [152–154]).

Lastly, all future research would certainly benefit from a more in-depth investigation of the precise neurophysiological mechanisms underlying the effects of cerebellar neurostimulation, which are currently not fully understood. Indeed, although cerebellar TMS induces both local as well as distal neurophysiological effects (see [155, 156]), driving synchronization of cerebello-cortical and cortico-cortical networks [157], it is still unclear which neural structures in the cerebellar cortex are most susceptible to stimulation [158]. It has been suggested that the modulation of cerebellar excitability involves long-term depression (LTD) and long-term potentiation (LTP) associated with local synaptic processes at the level of inhibitory Purkinje cells [158, 159]. However, another possibility is that Purkinje cells are stimulated trans-synaptically through parallel or climbing fibers [158], an option that deserves further consideration. Yet, evidence shows that TMS modulates cerebellar physiology also through facilitatory/inhibitory effects on excitatory granule cells and GABA-ergic interneurons [160]. Similarly, the electric field induced via cerebellar tDCS is suggested to polarize the superficial cortical layer that includes the large Purkinje cells [159, 161], but it is also likely to affect other neural elements in the cerebellar cortex, including granule and inhibitory cells, as well as climbing and mossy fibers, which explains why the direction of the physiological tDCS effect is difficult to predict [159]. Therefore, while cerebellar stimulation reliably induces behavioral and physiological modulation as evidenced by several controlled studies, including those presented here, further research is still required to appreciate the neurophysiological mechanisms at play, which would provide valuable insights to translate this knowledge into clinical applications for patients with hereditary ataxia.

## Conclusions

In recent years, the cerebellum has increasingly attracted scientists interested in basic and clinical research of neuromodulation. Cerebellar alterations are related to significant difficulties in the ability to regulate one’s affects and to infer and understand others’ mental states both in hereditary ataxia patients and in other clinical conditions. Nevertheless, research on potential treatments to improve socio-affective abilities in these populations is currently lacking. NIBS, including TMS and tDCS, have been deemed as an effective treatment strategy for several mental conditions (for a meta-analysis see [162]). Indeed, consistent evidence points to the efficacy of NIBS in treating core symptoms and cognitive functions in different neurological and neuropsychiatric disorders as well as in improving behavioral and socio-affective deficits (see [163, 164]). Thus, the manipulation of cerebro-cerebellar circuits through NIBS, by modulating behavior as well as cognitive and socio-affective functions, offers an opportunity to explore therapeutic interventions that could ameliorate cognitive and affective deficits in hereditary ataxia patients.

Despite the scant evidence applying cerebellar NIBS to boost socio-affective functions in this specific population, the studies reviewed here support the potential efficacy of different cerebellar neurostimulation protocols in modulating mentalizing functions in healthy individuals and reducing affective and social symptoms in neurologic and psychiatric conditions associated with cerebellar alterations. Based on the hypothesis that conceives the cerebellum as a *predictive device* in social and affective functions, such a beneficial effect may depend on the stimulation boosting the formation of internal models of physical and social events and the implicit learning of regularities in other individuals’ behavior. Future research should clarify the cerebello-cerebral networks causally involved in affective and social behavior as well as the specific functional operations implemented by these circuits.

## Data Availability

This declaration is not applicable.

## Abbreviations

ABC: Aberrant Behavior Checklist
ASD: Autism spectrum disorder
CCAS: Cerebellar Cognitive Affective Syndrome
CCASS: Cerebellar Cognitive Affective Syndrome Scale
CDSS: Calgary Depression Scale for Schizophrenia
CGI: Clinical Global Impressions scale
cTBS: Continuous theta-burst stimulation
dlPFC: Dorsolateral prefrontal cortex
dmPFC: Dorsomedial prefrontal cortex
GM: Gray matter
IAPS: International Affective Picture System
iTBS: Intermittent theta-burst stimulation
MADRS: Montgomery Asberg Depression Rating Scale
MRI: Magnetic resonance images
MT: Motor threshold
NIBS: Non-Invasive Brain Stimulation
PANAS: Positive and Negative Affect Schedule
PANSS: Positive and Negative Syndrome Scale
PHQ-9: Patient Health Questionnaire-9
POMS: Profile of Mood States
RMET: Reading the Mind in the Eyes test
rTMS: Repetitive Transcranial Magnetic Stimulation
SCA: Spinocerebellar ataxia
SF-36: Short Form Health Survey 36
SRT: Serial Reaction Time
STS: Superior Temporal Sulcus
tACS: Transcranial alternating current stimulation
TBS: Theta-burst stimulation
tDCS: Transcranial Direct Current Stimulation
TMS: Transcranial Magnetic Stimulation
ToM: Theory of Mind
TPJ: Temporoparietal junction
VAS: Visual Analogue Scale
